# Structure-informed clustering for population stratification in association studies

**DOI:** 10.1186/s12859-023-05511-w

**Published:** 2023-10-31

**Authors:** Aritra Bose, Myson Burch, Agniva Chowdhury, Peristera Paschou, Petros Drineas

**Affiliations:** 1 Computational Genomics, IBM T.J Watson Research Center, Yorktown Heights, NY USA; 2 Department of Computer Science, Purdue University, West Lafayette, IN USA; 3 Computer Science and Mathematics Division, Oak Ridge National Laboratory, Oak Ridge, TN USA; 4 Department of Biological Sciences, Purdue University, West Lafayette, IN USA

**Keywords:** Association studies, Populations structure, Clustering

## Abstract

**Background:**

Identifying variants associated with complex traits is a challenging task in genetic association studies due to linkage disequilibrium (LD) between genetic variants and population stratification, unrelated to the disease risk. Existing methods of population structure correction use principal component analysis or linear mixed models with a random effect when modeling associations between a trait of interest and genetic markers. However, due to stringent significance thresholds and latent interactions between the markers, these methods often fail to detect genuinely associated variants.

**Results:**

To overcome this, we propose CluStrat, which corrects for complex arbitrarily structured populations while leveraging the linkage disequilibrium induced distances between genetic markers. It performs an agglomerative hierarchical clustering using the Mahalanobis distance covariance matrix of the markers. In simulation studies, we show that our method outperforms existing methods in detecting true causal variants. Applying CluStrat on WTCCC2 and UK Biobank cohorts, we found biologically relevant associations in Schizophrenia and Myocardial Infarction. CluStrat was also able to correct for population structure in polygenic adaptation of height in Europeans.

**Conclusions:**

CluStrat highlights the advantages of biologically relevant distance metrics, such as the Mahalanobis distance, which captures the cryptic interactions within populations in the presence of LD better than the Euclidean distance.

**Supplementary Information:**

The online version contains supplementary material available at 10.1186/s12859-023-05511-w.

## Background

The basic principle underlying Genome Wide Association Studies (GWAS) is a test for association between genotyped variants for each individual and the trait of interest. GWAS have been extensively used to estimate the signed effects of trait-associated alleles and also map genes to disorders. Over the past decade, about 10,000 strong associations between genetic variants and one (or more) complex traits have been reported [1–3]. One unambiguous conclusion from GWAS is that for almost any complex trait that has been studied so far, genetic variation is linked with many loci contributing to the polygenic nature of the traits. Hence, on average, the proportion of variance explained at the single marker is very small [2].

One of the key challenges in GWAS are confounding factors, such as population stratification, which can lead to spurious genotype-trait associations [4, 5]. In subdivided populations, *linkage disequilibrium* (LD) is captured in two ways: the average LD in sub-populations owing to migrations and the covariance between concerned genetic loci capturing epistatic interactions [6]. Natural selection also plays a crucial role in association studies where in some cases selection can lead to allele frequencies being almost perfectly correlated with population structure [7]. Admixture of genetically distinct populations can generate LD throughout the genome [6] and hence it can lead to cause genuine genetic signals associated with a complex trait be mired in LD with related spurious loci. A related phenomenon, the so-called cryptic relatedness, is caused by individuals who are closely related and often grouped together by standard population structure correction strategies, and poses a serious confounding problem [8]. Two popular approaches for stratification correction while building the so-called *Genetic Relationship Matrix* (GRM) [9, 10] involve (i) including the principal components of the genotypes as adjustment variables [4, 11], and (ii) fitting a *Linear Mixed Model* (LMM) with an estimated kinship or GRM from the individual’s genotypes [1].

Recently, three independent studies [12–14] failed to replicate the previously reported signals of directional selection on height in European populations, as seen in the GIANT consortium (253,288 individuals [15]) in the independent and more recent UK Biobank cohort (500,000 individuals [16]). They further showed that the GIANT GWAS is confounded due to stratification along the north to south axis, where strong signals of selection were previously reported. These recent studies highlight the need for more sophisticated tools for correcting for population stratification.

Our work proposes a simple clustering-based approach to correct for stratification better than existing methods. As discussed above, it is important to consider the covariance matrix of genetic variants while constructing the GRM to account for the LD between genetic variants and synthetic LD due to population structure as potential confounders while performing association studies. This method takes into account the linkage disequilibrium while computing the distance between the individuals in a sample. Our approach, called CluStrat, performs *Agglomerative hierarchical clustering* (AHC) using a regularized Mahalanobis distance-based GRM, which captures the population-level covariance (LD) matrix for the available genotype data. We test CluStrat on large-scale simulated data of discrete and admixed, complex-structured populations with over one million genetics markers (Single Nucleotide Polymorphisms or SNPs for short). We observe that our approach identifies more less frequent variants at causal loci while maintaining low spurious associations when compared to standard stratification correction strategies across varying thresholds of significance. Computing the GRM by low-rank Mahalanobis distance, we apply CluStrat to large cohorts such as Wellcome Trust Case Control Consortium 2 (WTCCC2) and UK Biobank (UKBB) to find biologically significant associations in two complex diseases, namely Schizophrenia (SCZ) and Acute Myocardial Infarction (AMI) with potential variants implicated in the disease of interest which are often overlooked by GWAS. CluStrat also corrects for the uncorrected population structure in polygenic adaptation of height in Europeans, as highlighted in previous studies [12, 13]. Of independent interest is a simple, but not necessarily well-known, connection between the regularized Mahalanobis distance-based GRM that is used in our approach and the leverage and cross-leverage scores of the genotype matrix (see Methods and Additional file 1).

## Results

### Simulated data

We applied CluStrat to 100 simulation scenarios, modelling proportions of true genetic effect and admixture using three well-known models to generate simulated data: Balding-Nichols (BN) [17]; Pritchard-Stephens-Donnelly (PSD) [5]; and the 1000 Genomes project (TGP). We also used a “mosaic-chromosome” simulation scheme applied to British and Irish populations in the UK Biobank (UKBB model). We compared CluStrat’s performance with standard population structure correction approaches such as Eigenstrat [11] and PLINK2 [18]. We compared these methods on all 100 scenarios with the *p*-value threshold set to $5\times 10^{-8}$. We used GCTA tools [19] to simulate binary traits with 20% of the individuals as cases and enforcing 100 of the SNPs to be causal with heritability set to 0.5.

The BN and PSD model simulate scenarios with unrelated isolated populations. The PCA plot of the samples clearly show three isolated clusters with no connections between them in the BN model. In the PSD model, we see admixed populations between the clusters (see Additional file 1: Figs. S2 and S3). These data serve as our “base case” for arbitrarily structured populations with and without admixture. On the other hand, the TGP model is more realistic, drawing genotypes from allele frequency distributions from the 1000 Genomes Phase 3 dataset [20]. Projection of genotypes drawn from the 1000 Genomes (TGP) dataset on the top two axes of variations shows the distribution of samples across the world (Additional file 1: Figure S4). Additionally, the UKBB model is another more realistic simulation for admixture between British and Irish populations (Additional file 1: Figure S5).

**Fig. 1:**
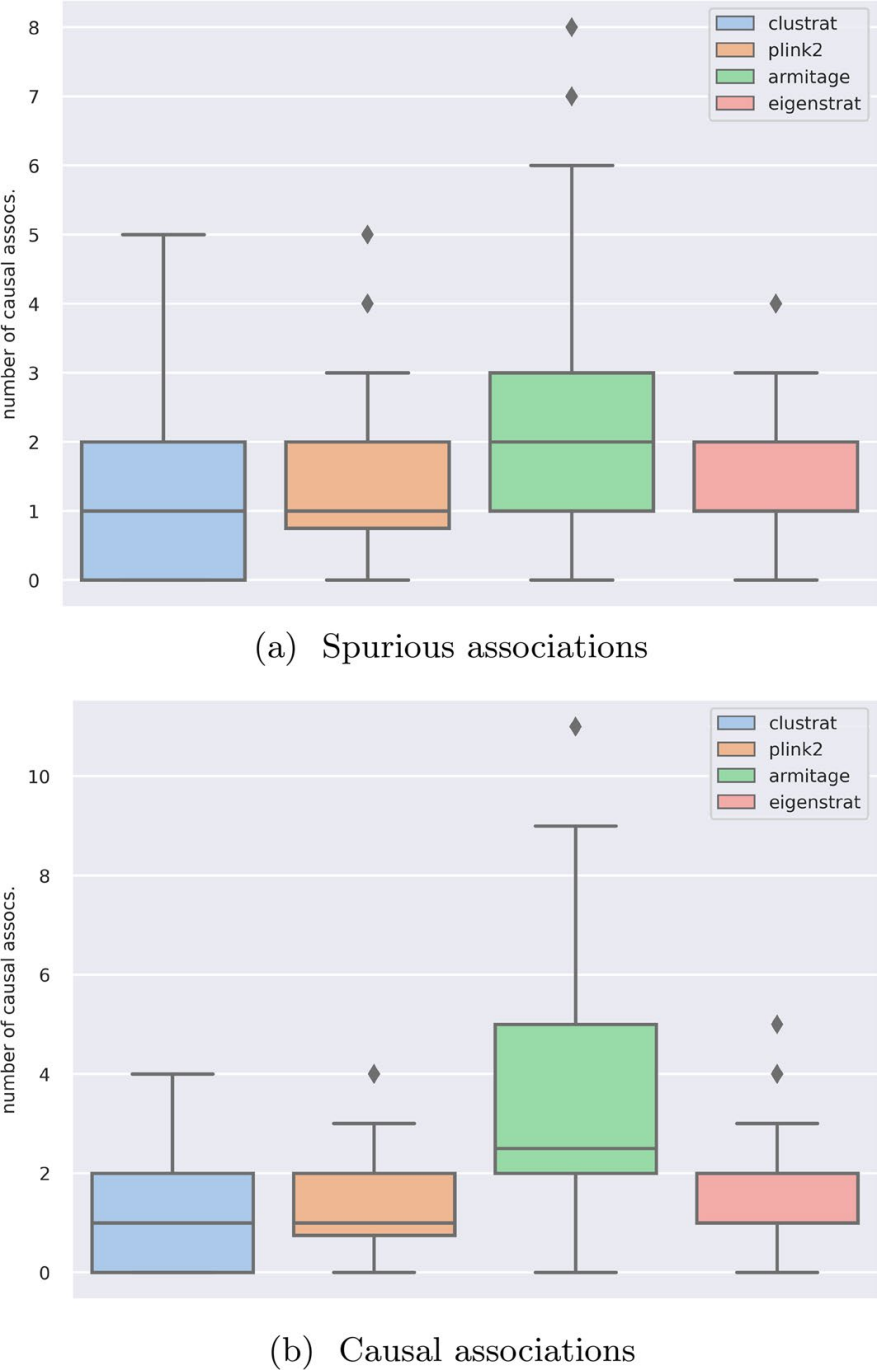
Box plots for spurious and causal associations on the PSD model using the CluStrat, PLINK2, Armitage trend $\chi ^2$ statistic, and Eigenstrat

The Armitage trend $\chi ^2$ test with no population structure correction returns many of the SNPs in the simulation study as true associations. This results in more spurious associations, clearly highlighting the need for population structure correction. PCA or LMM based approaches return roughly the expected number of spurious associations, as also shown in prior work [11]. CluStrat increases the number of detected causal variants over standard approaches. The Armitage trend $\chi ^2$ test returns the maximum number of causal associations, but also results in the largest number of spurious associations. CluStrat outperforms all other standard methods for population stratification correction in this scenario, without returning any spurious associations (Fig. 1).

*Correcting for population stratification in the height GWAS* To assess whether CluStrat accurately corrects for previously found uncorrected population stratification [12] in polygenic adaptation of alleles associated with height in Europeans, we applied it on the UKBB cohort. We assessed the singleton density scores (SDS), which use a coalescent approach to infer recent changes in allele frequencies from contemporary genome sequences [21]. SDS was combined with GWAS effect size estimates to infer polygenic adaptation of complex traits, generating a *tSDS score* [12], by assigning the SDS sign to the trait-increasing allele. A tSDS score larger than zero for height-increasing alleles implies that these alleles are increasing in frequency in a population over time due to natural selection [12].

**Fig. 2:**
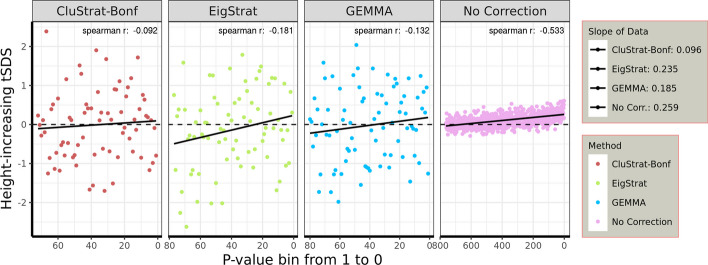
tSDS for height-increasing alleles in the UK Biobank subset using Bonferonni corrected CluStrat, the PCA-based Eigenstrat method, and the LMM-based GEMMA method. SNPs are ordered by *p*-value (in bins of 50 in the ’No correction’ scenario). The dashed line indicates null-expectation and the black line is the linear regression fit

We used 18,698 highly-related individuals in the UK Biobank cohort (first degree or higher according to the kinship coefficient) genotyped on 44,818 SNPs, related to the largest effect sizes in relation to height, from summary statistics data generated by [15]. We found that CluStrat corrects for underlying population structure with a slope between the height-increasing tSDS and the *p*-values of SNPs obtained from CluStrat close to zero (0.096). The linear regression fit for CluStrat is almost identical to the null-expectation. We also found that the height-increasing tSDS and the *p*-values from CluStrat have a negligible Spearman’s correlation coefficient ($r=-0.092$ and $p = 0.664$). Therefore, there is no monotonic association between the height-increasing tSDS and the association test *p*-values obtained from CluStrat. Similar to the simulation scenarios, CluStrat ends up selecting a similar number of SNPs with other methods such as PCA-based Eigenstrat and LMM-based GEMMA (Fig. 2). However, the markers with a polygenic effect on the trait under investigation reach significance and are responsible for better population stratification correction.

### Real data

We applied CluStrat on data from two complex diseases: SCZ data from WTCCC2’s and AMI data from the UK biobank. In both cases, CluStrat identified biologically relevant associations.

**Fig. 3:**
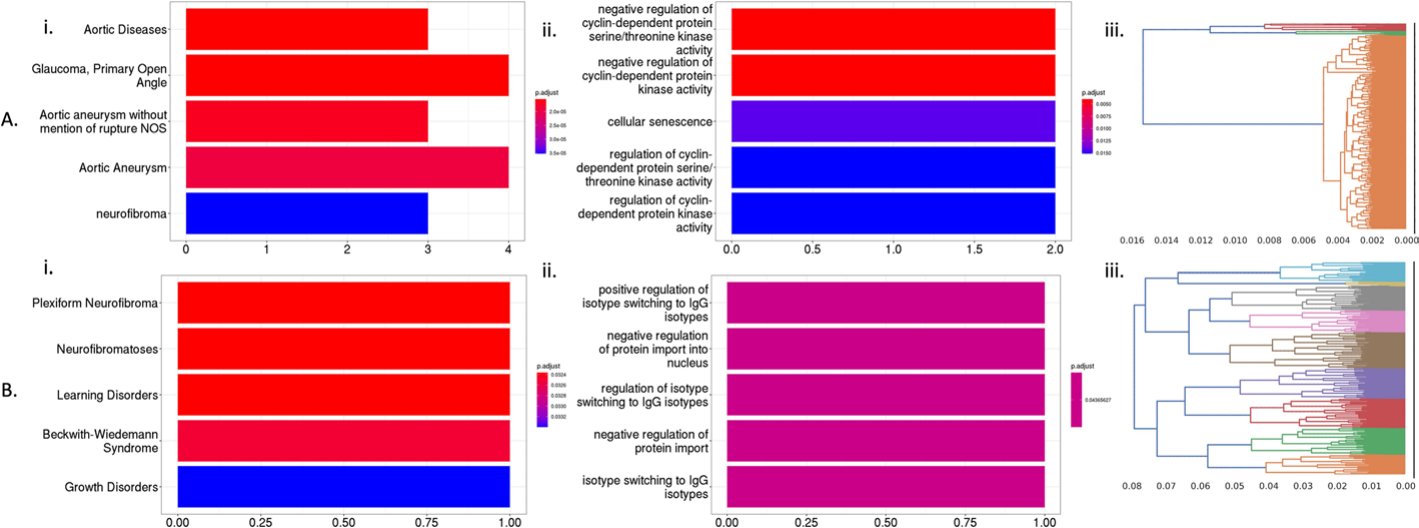
Applying CluStrat on **A** AMI and **B** SCZ data. Bar plot of significantly ($p < 5\times 10^{-8}$) enriched pathways showing cellular functions from (i) DOSE and (ii) GO databases. Bars are colored by *p*-values and the *x*-axis denotes the number of genes found in the pathway. (iii) Dendrogram obtained after applying Agglomerative Hierarchical Clustering (AHC) is colored by the number of clusters and shows the depth of the branches in the *x*-axis

*CluStrat corrected SCZ SNPs* We applied CluStrat using two clusters on SCZ data and identified 5 variants with a *p*-value threshold of $5\times 10^{-8}$. These variants map to significantly enriched pathways such as *neurofibroma* in the DOSE database; *immunoglobulin isotypes (IgG)* in GO (Fig. 3). These pathways are directly associated with the incidence of SCZ. Upon further investigation, many of these CluStrat-corrected variants mapped to genes relevant to SCZ including FAM83B and CABP1 (see Additional file 1 for details).

We applied CluStrat after pruning for LD in the original data with correlation ($r^2$) thresholds of 0.9 and 0.2, to showcase its performance in low LD scenarios. We show that we could replicate all 7 and 4 of the 7 top significantly associated markers when using $r^2 = 0.9$ and $r^2 = 0.2$, respectively. These variants were exactly replicated when we applied clumping with the same $r^2$ thresholds to the non-pruned data. We further performed annotation of the associations using GWAS catalog data and obtained *sporadic Amyotrophic lateral sclerosis* as the only previously associated traits with these markers and. All of which were also replicated when we applied CluStrat on the pruned data (Additional file 1: Figure S8).

*CluStrat corrected AMI SNPs* We applied CluStrat using two clusters and identified 26 variants with a *p*-value threshold of $5\times 10^{-8}$. The identified variants are significantly over-represented in biological pathways such as *aortic diseases* and *aortic aneurysms* in the DOSE database; *kinase activity* and *cellular senescence* in GO. All of these pathways are directly associated with the incidence of AMI. Upon further investigation, many of these CluStrat-corrected variants mapped to genes relevant to AMI including CDKN2B, ATXN2 and LDLR. (See Additional file 1 for details)

We applied CluStrat after pruning for LD in the original data with correlation ($r^2$) thresholds of 0.9 and 0.2, to showcase its performance in low LD scenarios. We show that we could replicate 16 and 3 of the 26 top significantly associated markers when using $r^2 = 0.9$ and $r^2 = 0.2$, respectively. These variants were exactly replicated when we applied clumping with the same $r^2$ thresholds to the non-pruned data. We further performed annotation of the associations using GWAS catalog data and obtained *coronary artery disease*, *open angle glaucoma*, *body mass index*, *systolic blood pressure*, *type II diabetes mellitus*, etc. as the previously associated traits with these markers. All of which were also replicated when we applied CluStrat on the pruned data (Additional file 1: Figure S9).

## Discussion

CluStrat provides a structure informed clustering approach to correct for population stratification in GWAS. In our experiments, we verified the power of our approach in a variety of simulated data and observed that CluStrat outperforms the widely used Eigenstrat and PLINK2 methods in all settings, by detecting more causal SNPs and almost no spurious associations. This shows that structure informed clustering of the genotype data by using Mahalanobis distance followed by regularized association tests robustly outperforms genotype and phenotype adjustments using the top principal components, which is what PCA and LMM-based methods typically do. We chose the low-rank Mahalanobis distance metric in CluStrat because it captures the LD-induced structure information in the GRM. We established a link between the low-rank Mahalanobis distance and the low-rank leverage/cross-leverage scores, which allows us to get around the storage and computational bottlenecks of Mahalanobis distance. Prior work [22] computed the Mahalanobis distance by randomly sub-sampling a small number of SNPs to estimate the covariance matrix and circumvent the computational time and space requirements. Mahalanobis distance is also shown to remove bias in heritability estimates in the presence of LD, therefore finding true causal variants [23]. We showed that the Mahalanobis distance performs better (Additional file 1: Figure S6) in capturing cryptic relatedness compared to the Euclidean-distance-based GRM. CluStrat is not sensitive to the number of clusters as we employ a five-fold cross validation scheme to obtain the optimal number of clusters for each data set. See Additional file 1 for details.

PCA-based methods have been under scrutiny recently as independent studies [12, 13] on the UKBB [16] failed to replicate the genetic associations of heritable height in Europeans, where a positive selection signal was observed in a north to south gradient [24, 25] in the GIANT [15] cohort. These studies attributed the failure to replicate the results to cryptic relatedness among individuals, which PCA-based approaches for population stratification correction do not always correct. CluStrat provides a fine structure-based clustering approach to tackle cryptic relatedness and ancestral differences among the individuals between and within populations. Importantly, it corrects for population stratification in height GWAS almost perfectly. CluStrat was applied on the same data set as used in previous studies showing that the polygenic adaptation of height along the north to south gradient in Europe was overestimated [12]. CluStrat has the smallest slope with the same direction as others methods in tSDS scores for the height-increasing alleles in the UK Biobank dataset, while selecting almost the same number of SNPs as Eigenstrat and GEMMA. CluStrat achieves almost perfect correction, with negligible correlation between the pre-computed tSDS and the actual *p*-values.

Applying CluStrat to complex diseases, such as SCZ and AMI, we found novel variants and replicated previously associated SNPs/genes with these diseases. In SCZ, pathways such as *immunoglobulin isotypes (IgG)* and *neurofibroma* were identified as significantly enriched enriched in the CluStrat-corrected SNPs. SCZ is characterized by an interrelated activation of the immune-inflammatory response system and there is established evidence of *immunoglobulin’s* role in the immune response [26]. *Neurofibromatosis* (NF) is often associated with neurodevelopmental disorders, which are more frequent in NF than in general population [27]. In AMI, pathways related to *aortic diseases*, *aortic aneurysms*, *kinase activity*, and *cellular senescence* were shown to be significantly enriched in the CluStrat-corrected SNPs. *Aortic aneurysms* occur when the aorta weakens and bulges. Ruptures of this vessel can cause life-threatening bleeding. These types of aneurysms can also force blood away from organs and tissues, leading to AMI. *Protein kinases* are intimately involved in different signal pathways for the regulation of cardiac function to maintain healthy cardiac function, but also participate in the development of cardiac dysfunction in AMI and heart failure  [28]. *Cellular senescence* has received recent attention as a potential target preventing cardiovascular diseases [29]. The amount of senescent cells in an individual’s body increases with age and as the aging immune system becomes less efficient, senescent cells accumulate and taint healthy cells. This can affect a person’s ability to prevent illness such as cardiovascular diseases.

The power of CluStrat is further revealed when we pruned for LD in the genotype data after QC with differing $r^2$ thresholds to reflect whether CluStrat can work in conditions of low LD. We observe that both in SCZ and AMI traits, CluStrat overwhelmingly recovered most significant SNPs from the pruned genotypes with $r^2 = 0.9$ and a handful of the top-most significant markers with a stringent threshold for pruning ($r^2=0.2$). Interestingly, it could capture almost all of the previously mapped traits in GWAS catalog, demonstrating that even in low LD scenarios, CluStrat correctly obtains the most significant markers when compared with the performance on non-pruned genotype data providing further support for doing LD-based GRM computation and population structure correction.

## Conclusions

In summary, CluStrat highlights the advantages of biologically relevant distance metrics, such as the Mahalanobis distance, which captures the cryptic interactions within populations in the presence of LD better than the Euclidean distance. We evaluated CluStrat on multiple simulated data for arbitrarily structured populations with and without admixture. We concluded that CluStrat outperforms PCA or LMM based population stratification correction techniques in a variety of simulated datasets. CluStrat accurately corrected for population stratification in height GWAS in UKBB and identified numerous previously annotated genes and pathways for SCZ and AMI, as well as novel candidate loci. Thus, structure informed clustering of genetic data can remove cryptic population stratification in association studies and can be used to mitigate confounding in polygenic risk scores and precision medicine initiatives.

## Methods

### Notation

Let $\textbf{X}\in \mathbb {R}^{m \times n}$ denote the genotype matrix (e.g., the minor allele frequency (MAF) matrix on *m* samples genotyped on *n* SNPs). The matrix is appropriately normalized as is common in population genetics analyses to have zero mean and variance one (columnwise). The vector $y \in \mathbb {R}^m$ represents the trait of interest and its *i*-th entry is set to one for cases and to zero for controls (for binary traits). We let $\textbf{X}_{i*}$ denote the *i*-th row of the matrix $\textbf{X}$ as a row vector and $\textbf{X}_{*i}$ denote the *i*-th column of the matrix $\textbf{X}$ as a column vector. We represent the top *k* left singular vectors of the matrix $\textbf{X}$ by the matrix $\textbf{U}_k \in \mathbb {R}^{m \times k}$ and we will use the notation $(\textbf{U}_k)_{i*}$ to denote the *i*-th row of $\textbf{U}_k$ as a row vector.

### CluStrat

CluStrat provides an LD based clustering framework to capture the population structure and the tests for association within each cluster, as described in Algorithm 1.
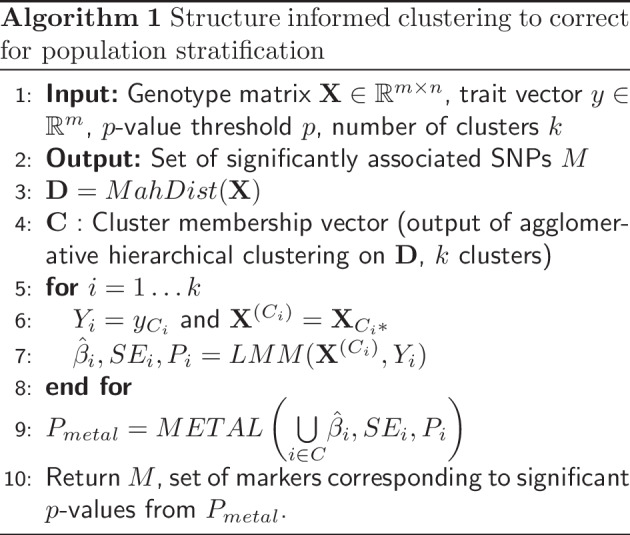


The algorithm computes the distance matrix $\textbf{D}$ from the normalized genotype matrix $\textbf{X}$ and performs AHC for a number of clusters *k*, selected using five-fold cross validation. We perform the association test in CluStrat by using linear models (logistic or linear regression based on the input) on each cluster. Then, we take the results for each cluster and perform meta-analysis, using METAL [30], improving the power to detect associations.

### Mahalanobis distance based GRM

We now briefly discuss the use of the Mahalanobis distance at the first step of the proposed algorithm. In an arbitrarily structured breeding population, correlation between loci due to LD often results in block-diagonal structures in the covariance matrix of genetic variants. Thus, it is important to account for this LD structure in the computation of the distance matrix [22]. One way to account for the LD structure is to use the squared Mahalanobis distance [31, 32] (denoted as $\textbf{D}$ in eqn. 1). Given a matrix $\textbf{G}\in \mathbb {R}^{n \times n}$ which contains the covariance structure of LD (covariance due to LD between genetic markers), the LD-corrected GRM implementing the Mahalanobis distance is defined as

1 $$\begin{aligned} \textbf{D}= \textbf{X}\textbf{G}^{-1} \textbf{X}^{\top }. \end{aligned}$$

The Mahalanobis distance is useful in high-dimensional settings where the Euclidean distances fail to capture the true distances between observations (see Additonal File 1 for relationships between Mahalanobis and Euclidean distances). It achieves this by taking the correlation structure between the features into account.

### Computing the Mahalanobis distance

The Mahalanobis distance is known to be connected to statistical leverage [33]. We discuss the connection between a regularized version of the Mahalanobis distance and a regularized notion of statisical leverage scores below. We first note that the Mahalanobis distance is invariant to linear transformations, which means that the standard normalizations of the genotype matrix $\textbf{X}$ do not affect the Mahalanobis distance between two vectors. Recall the definition of the Mahalanobis distance between samples *i* and *j*:

2 $$\begin{aligned} \textbf{D}(\textbf{X}_{i*}, \textbf{X}_{j*}) = (\textbf{X}_{i*}-\textbf{X}_{j*})\textbf{G}^{-1}(\textbf{X}_{i*}-\textbf{X}_{j*})^{\top }. \end{aligned}$$

Now, recall that the rank-*k* leverage scores of the genotype matrix $\textbf{X}\in \mathbb {R}^{m \times n}$ with $n \gg m$ are defined by the row norms of the matrix of its top *k* left singular vectors $\textbf{U}_k \in \mathbb {R}^{m \times k}$. Let $(\textbf{U}_k)_{{i*}}$ denote the *i*-th row of the matrix $\textbf{U}_k$. Then the rank-*k* statistical leverage scores of the rows of $\textbf{A}$, for $i =1,\ldots ,n$ are given by $\textbf{H}_i = \Vert (\textbf{U}_k)_{{i*}} \Vert _2^2$. Similarly, the rank-*k* (*i*, *j*)-th cross-leverage score, $\textbf{H}_{ij}$, is equal to the dot product of the *i*-th and *j*-th rows of $\textbf{U}_k$, namely

3 $$\begin{aligned} \textbf{H}_{ij} = \langle (\textbf{U}_k)_{{i*}}, (\textbf{U}_k)_{{j*}} \rangle . \end{aligned}$$

Here, $\textbf{H}\in \mathbb {R}^{m \times m}$ is the matrix of all leverage and cross-leverage scores. We note that $\textbf{H}_i = \textbf{H}_{ii} = \Vert (\textbf{U}_k)_{{i*}} \Vert _2^2 = \left( \textbf{U}_k\textbf{U}_k^{\top }\right) _{ii}$ is a special case of the dot product in eqn. 3 for the diagonal leverage scores. We show that the Mahalanobis distance can be written in terms of the rank-*k* leverage and cross-leverage scores (see Additional file 1 for details on the relationship between Mahalanobis distance and leverage scores). Indeed, the final formulas are:

4 $$\begin{aligned} \textbf{D}_{i} = \textbf{D}(\textbf{X}_{i*},0)&= (m-1)\left( \textbf{H}_{i}-{1}/{m}\right) , \text{ and } \end{aligned}$$

5 $$\begin{aligned} \textbf{D}_{ij} = \textbf{D}(\textbf{X}_{i*}, \textbf{X}_{j*})&= (m-1)(\textbf{H}_{i} + \textbf{H}_{j} - 2\textbf{H}_{ij}). \end{aligned}$$

Thus, we show that the Mahalanobis distance between two vectors can be computed efficiently without storing or inverting $\textbf{G}$, by the corresponding rank-*k* leverage and cross-leverage scores. By computing the rank-*k* Mahalanobis distance with respect to the top *k*-left singular vectors of the genotype matrix $\textbf{X}$, we make this computation feasible for UK Biobank-scale datasets using methods such as TeraPCA [34] to approximate the matrix $\textbf{U}_k$ accurately and efficiently.
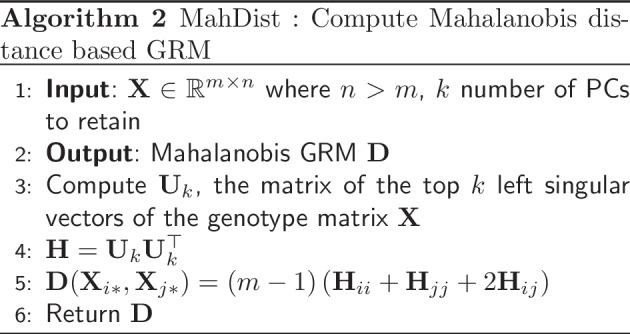


### Agglomerative hierarchical clustering (AHC)

We performed AHC using the LD induced Mahalanobis distance with a varying number of clusters. We set the expected number of clusters to $d + q$ where *d* is the number of populations in the data and *q* is a user-defined range. We performed a five-fold cross-validation to choose the optimal number of clusters and retain the cluster which maximizes the intersection of associations across all the clusters. The observed number of clusters is obtained by the inconsistency method of pruning according to the depth of the dendrogram. We note that for the simple case where *q* is set to zero, the clustering essentially attempts to recover the populations. In practice, we observed that the number of qualitative clusters obtained by running PCA on the genotype data serves as a good heuristic for the number of user defined clusters using the AHC procedure.

### Data

*Simulated Data.* We generated an extensive set of simulations with challenging scenarios to demonstrate the robustness to different real-world scenarios and power to detect few spurious associations.

For the genotype data, we simulated allele frequencies using *(i)* Balding-Nichols (BN) model [17] based on allele-frequency and $F_{ST}$ estimates calculated on the HapMap data set; *(ii)* different levels of admixture by varying the parameter $\alpha$ in the Pritchard-Stephens-Donnelly model (PSD) [5]; *(iii)* structure estimated from 1000 Genomes Project (TGP) [20] (see Additional file 1 for details); and a “mosaic-chromosome” simulation scheme applied to British and Irish populations in the UK BioBank (UKBB) [35, 36]. For the phenotype data, we used GCTA tools [19] that employ a simple additive genetic model to create a synthetic trait based on the simulated genotype data. We also enforced 20% of the simulated individuals to be cases and the remainder to be controls. These tools allow us to control heritability of liability and disease prevalence for the generated phenotype.

*Real data* To capture real world population structure, we applied CluStrat on two complex diseases: SCZ and AMI. SCZ data was available from the Wellcome Trust Case Control Consortium (WTCCC2) study containing 5893 individuals (5416 SCZ controls and 477 cases) with 18,683 markers after performing quality control (QC) using PLINK v2 [37]. We also applied on AMI data from the UK Biobank (UKBB) with 23,142 individuals (11,610 controls and 11,532 cases) and 208,337 genotypes after QC.

On the genotypes passing QC, we applied CluStrat before and after pruning for LD to showcase the utility of considering the genotype covariance matrix while correcting for LD due to population structure and epistatic effects. We used multiple correlation ($r^2$) thresholds of 0.9 and 0.2 to compare summary statistics of a relaxed and stringent threshold, respectively.

### Pathway analysis

We performed pathway analysis for clusterProfiler v3.10.1 [38] using pathways from Disease Ontology Semantic and Enrichment (DOSE), Gene Ontology (GO), and Kyoto Encyclopedia of Genes and Genomes (KEGG) databases.

### Variant annotation

We annotated the Clustrat-corrected variants using Ensembl Variant Effect Predictor (VEP) [39]. We used LOFTEE [40] for annotating loss-of-function (LoF) variants. We used the GWAS catalog [41] to map the variants to associated traits from the catalog. We used DisGeNET [42] to obtain the disease-gene pairs for SCZ and AMI and mapped them with CluStrat-corrected genes.

### Supplementary Information

**Additional file 1:** Methods related to CluStrat including theoretical background and proof and additional results of different simulation scenarios and real data from WTCCC2 and UKBB.

## Data Availability

Code is available at https://github.com/aritra90/CluStrat.

## Abbreviations

GWAS: Genome-wide Association Studies
LD: Linkage disequillibrium
GRM: Genetic Relationship Matrix
LMM: Linear Mixed Model
AHC: Agglomerative Hierarchical Clustering
WTCCC2: Wellcome Trust Case Control Consortium 2
SCZ: Schizophrenia
AMI: Acute Myocardial Infarction
LOF: Loss of Function
BN: Balding-Nichols
PSD: Pritchard-Stephens-Donnelly
TGP: 1000 Genomes
PCA: Principal Component Analysis
SDS: Singleton Density Scores
MAF: Minor Allele Frequency
RLA: Randomized Linear Algebra
OR: Odds Ratio
DOSE: Disease Ontology Semantic and Enrichment
GO: Gene Ontology
KEGG: Kyoto Encyclopedia of Genes and Genomes
VEP: Variant Effect Predictor
