# Supplementary material for: Structure-informed clustering for population stratification in association studies
Source: BMC Bioinformatics. 2023 Oct 31;24:411. doi: 10.1186/s12859-023-05511-w (PMC10619291; doi:10.1186/s12859-023-05511-w)
Supplement: Supplementary file 1 — Additional file 1: Methods related to CluStrat including theoretical background and proof and additional results of different simulation scenarios and real data from WTCCC2 and UKBB. [file 12859_2023_5511_MOESM1_ESM.pdf]

Structure informed clustering adjusts for population stratification in association studies

Aritra Bose[1,†], Myson C. Burch[2,†], Agniva Chowdhury[3], Peristera Paschou[4], and Petros Drineas[2,*]

[1]IBM Research, Yorktown Heights, NY 10598.
[2]Computer Science Department, Purdue University, West Lafayette, IN 47907.
[3]Computer Science and Mathematics Division, Oak Ridge National Laboratory, Oak Ridge, TN 37830.
[4]Department of Biological Sciences, Purdue University, West Lafayette, IN 47907.
[†]Equal Contribution
[*]Corresponding authors: pdrineas@purdue.edu

Supplementary Note

Simulated Data

Our simulation study on quantitative traits with population structure as a latent variable is constructed in five different ways for three different proportions of variance for genetic effects, non-genetic effects, and random noise, all of which contribute to the trait. We simulated 100 independent datasets containing $m = 1,000$ individuals and $n = 100,000$ markers from a quantitative trait model 1. Let Z be a latent variable which captures environmental factors that are affected by population structure. Equation 1 allows interdependence of structure, lifestyle and environment. We assume $\mathbf{E}[\epsilon_j|z_j] \sim \mathcal{N}(0, \sigma^2(z_j))$ allowing for heteroskedasticity of the random noise variation [18]. Therefore, $x^j = (x_{1j}, x_{2j}, \cdots, x_{mj})^\top$, while λ_j and σ^2 can be thought of as functions of z_j, where $Z = (z_1, z_2, \cdots, z_m)$. λ_j is unspecified but along with z_j, they are assumed to be dependent, random variables. Thus, the population genetic model is dependent on the structural variable z_j for each individual. We define the corresponding binary trait model as

$$\log\left(\frac{\Pr(y_j = 1)}{\Pr(y_j = 0)}\right) = \alpha + \sum_{i=1}^{m} \beta_i x_{ij} + \lambda_j \tag{1}$$

using the Odds Ratio (OR) as the classifier for disease status from the continuous variable y. We set $\mathbf{Var}\left[\sum_{i=1}^{n} \beta_i x_{ij}\right]$, $\mathbf{Var}\left[\sum_{j=1}^{n} \lambda_j\right]$, and $\mathbf{Var}[\epsilon_j]$ to (5%,5%,90%), (10%,0%,90%), and (10%,20%,70%), respectively, using all possible combinations. Thus, we varied the amount of genetic contribution to the trait for each simulation scenario, capturing variable amounts of population structure. We simulated ten truly associated SNPs, whose effect sizes were distributed according to a normal distribution and we set $\beta_i = 0$ for all other non-causal SNPs.

The genotype matrix $\mathbf{X} \in \mathbb{R}^{m \times n}$ consisting of the simulated allele frequencies was generated using the algorithms of [10,18]. More specifically, we set $\mathbf{F} = \mathbf{TS}$, where $\mathbf{T} \in \mathbb{R}^{m \times d}$ and $\mathbf{S} \in \mathbb{R}^{d \times n}$,

where $d \leq n$ is the number of population groups. $\mathbf{S}$ is the indicator matrix that encapsulates structure with n individuals and contained in d populations. On the other hand, $\mathbf{T}$ characterizes how the structure is manifested in the allele frequencies of each SNP [10]. Finally, projecting $\mathbf{S}$ onto the column space of $\mathbf{T}$, we obtain the allele frequency matrix $\mathbf{F}$. We sample $\mathbf{X}$ as a special case of $\mathbf{F}$ for Balding-Nichols (BN), Pritchard-Stephens-Donelly (PSD), and TGP (1000 Genomes Project) models, respectively. We formed $\mathbf{T}$ and $\mathbf{S}$ for the above five simulations with three scenarios each and continuous traits, resulting in 15 different evaluation scenarios for continuous and binary traits. The algorithm for constructing $\mathbf{T}$ and $\mathbf{S}$ is detailed in reference [10, 18].

For BN, the allele frequency matrix is simulated from the HapMap phase 3 dataset using three unrelated populations, CEU (Europeans), ASW (African ancestry in southwestern USA), and MEX (Mexicans) with the population differentiation due to genetic structure (F_{st}) set to 0.01. The final genotype matrix, $\mathbf{X}$, is drawn independently at random from the binomial distribution with the parameter n set to two, denoting the allele status (0,1 or 2) corresponding to homozygous major/minor or heterozygous; the probability p is set to the simulated allele frequency for each individual SNP. For PSD, the allele frequency matrix was drawn from the BN frequency distribution with the same populations. We simulate $\mathbf{S}$ using i.i.d draws from the Dirichlet distribution with varying values of α, which denotes the parameter influencing the relatedness between the individuals. We show results for $\alpha = \{0.01, 0.1, 0.5\}$.

Distance metrics for Hierarchical clustering

CluStrat computes the distance matrix $\mathbf{D}$ from $\mathbf{X}$ to perform the AHC. The choice of distance metric is user defined. However, we choose the distance metric based on LD induced distances to capture the cryptic relatedness between individuals in a population which is not otherwise captured by other stratification correction methods. We use the normalized genotype matrix $\mathbf{X}$ following the standard normalization procedure by minor allele frequency of each marker. Let us consider the unscaled GRM which captures the Euclidean distances as $\mathbf{D} = \mathbf{X}\mathbf{X}^\top$ and let $\mathbf{I} \in \mathbb{R}^{n \times n}$ be the identity matrix, where n is the number of markers. Thus $\mathbf{D}$ can be rewritten as

$$\mathbf{D} = \mathbf{X}\mathbf{I}\mathbf{X}^\top. \tag{2}$$

Thus, the identity matrix in the above expression captures un-correlatedness between all markers (same weighting on the diagonal for all markers). In an arbitrarily structured breeding population, there exists correlation between loci due to linkage. As a result, the identity matrix should be replaced by a different diagonal or, better, a block-diagonal matrix in the above expression. It is clearly important to account for this LD covariance structure in the computation of the GRM [15]. One way to account for the LD structure in GRM is to use the squared Mahalanobis distance [14, 16], denoted by $\mathbf{D}$. Given a matrix $\mathbf{G} \in \mathbb{R}^{n \times n}$ which contains the covariance structure of LD across all markers, the LD-corrected GRM using the Mahalanobis distance is defined as

$$\mathbf{D} = \mathbf{X}\mathbf{G}^{-1}\mathbf{X}^\top. \tag{3}$$

The RHS of equation 3 represents the squared multivariate Mahalanobis distance between individuals. The Mahalanobis distance is useful in high-dimensional settings where the Euclidean distance fails to capture the true distance between observations. It achieves this by computing the correlation between the features as captured by the SNP covariance matrix into account. The Cholesky factorization of the covariance matrix $\mathbf{G} = \mathbf{L}\mathbf{L}^\top$, where $\mathbf{L}$ is the lower diagonal

matrix known as the Cholesky factor of $\mathbf{G}$. We can rewrite equation 3 as

$$\begin{aligned}
\mathbf{X}\mathbf{G}^{-1}\mathbf{X}^\top &= \mathbf{X}\left(\mathbf{L}\mathbf{L}^\top\right)^{-1}\mathbf{X}^\top \\
&= \mathbf{X}(\mathbf{L}^\top)^{-1}(\mathbf{L})^{-1}\mathbf{X}^\top \\
&= \left(\mathbf{X}(\mathbf{L}^{-1})^\top\right)\left(\mathbf{L}^{-1}\mathbf{X}\right)^\top \\
&= \left(\mathbf{L}^{-1}\mathbf{X}^\top\right)^\top\left(\mathbf{L}^{-1}\mathbf{X}^\top\right) \\
&= \mathbf{Q}^\top\mathbf{Q}.
\end{aligned}$$

Here $\mathbf{Q} = \mathbf{L}^{-1}\mathbf{X}^\top$ represents the transformed variables and $\mathbf{Q}^\top\mathbf{Q}$ is the squared Euclidean distance between the transformed variables. Thus, the Mahalanobis distance accounts for the covariance between variables by transforming the data into an "uncorrelated form" and computing the euclidean distances between them.

Mahalanobis Distance and Leverage Scores

The Mahalanobis distance is known to be connected to statistical leverage [20] or leverage scores. We show this relationship by first noting that the Mahalanobis distance is invariant to linear transformations, which means that the Mahalanobis distance between two vectors

$$\mathbf{D}(\mathbf{X}_{i*}, \mathbf{X}_{j*}) = (\mathbf{X}_{i*} - \mathbf{X}_{j*})\mathbf{G}^{-1}(\mathbf{X}_{i*} - \mathbf{X}_{j*})^\top \tag{4}$$

could have zero mean for each vector. In our genotype matrix, $\mathbf{X} \in \mathbb{R}^{m \times n}$, we have n markers and m observations. The design matrix $\mathbf{X}$ on which we intend to fit the model must contain an intercept. Thus, we use $\mathbf{X}$ here to denote the design matrix including the intercept column, followed by one column for each SNP for all m individuals. Furthermore, as we compute the Mahalanobis distance with respect to the *low-rank* genotype matrix $\mathbf{X}_k$, we only consider the *low-rank leverage scores* (rather than the leverage scores of the original matrix $\mathbf{X}$), which are essentially the diagonal elements of the following projection matrix:

$$\mathbf{H} = \mathbf{X}_k\left(\mathbf{X}_k^\top\mathbf{X}_k\right)^{-1}\mathbf{X}_k^\top. \tag{5}$$

Similarly, the off-diagonal elements of $\mathbf{H}$ are called *cross-leverage scores* for $\mathbf{X}_k$. We now present a connection between the Mahalanobis distance and the leverage and cross-leverage scores. First, consider the diagonal elements of $\mathbf{H}$ $(i = j)$:

$$\mathbf{H}_{ii} = (1; \mathbf{X}_{k_{i*}})\left(\mathbf{X}_k^\top\mathbf{X}_k\right)^{-1}(1; \mathbf{X}_{k_{i*}})^\top. \tag{6}$$

Exploiting the structure of $\left(\mathbf{X}_k^\top\mathbf{X}_k\right)^{-1}$, we can reformulate it in terms of a block matrix as follows:

$$\mathbf{X}_k^\top\mathbf{X}_k = m\begin{pmatrix} 1 & \mathbf{0}^\top \\ \mathbf{0} & \mathbf{C} \end{pmatrix},$$

where $\mathbf{C}_{ij} = \frac{1}{m}\sum_{\ell=1}^{m}\mathbf{X}_{k_{\ell i}}\mathbf{X}_{k_{\ell j}} = \frac{m-1}{m}Cov(\mathbf{X}_{k_{*i}}, \mathbf{X}_{k_{*j}}) = \frac{m-1}{m}\mathbf{\Sigma}_{ij}$. Here $\mathbf{\Sigma}$ is the corresponding sample covariance matrix. Thus,

$$\left(\mathbf{X}_k^\top\mathbf{X}_k\right)^{-1} = \frac{1}{n}\begin{pmatrix} 1 & \mathbf{0}^\top \\ \mathbf{0} & \mathbf{C}^{-1} \end{pmatrix} = \begin{pmatrix} \frac{1}{n} & \mathbf{0}^\top \\ \mathbf{0} & \frac{1}{n-1}\mathbf{\Sigma^{-1}} \end{pmatrix}.$$

Using Equation 6 we get

$$\mathbf{H}_i = (1; \mathbf{X}_{k_{i*}}) \begin{pmatrix} \frac{1}{m} & \mathbf{0}^\top \\ \mathbf{0} & \frac{1}{m-1}\mathbf{\Sigma^{-1}} \end{pmatrix} (1; \mathbf{X}_{k_{i*}})^\top \tag{7}$$

$$= \frac{1}{m} + \frac{1}{m-1}\mathbf{X}_{k_{i*}}\mathbf{\Sigma^{-1}}\mathbf{X}_{k_{i*}}^\top \tag{8}$$

$$= \frac{1}{m} + \frac{1}{m-1}\mathbf{D}\left(\mathbf{X}_{k_{i*}}, 0\right). \tag{9}$$

Solving for $\mathbf{D}_i = \mathbf{D}(\mathbf{X}_{k_{i*}}, 0)$ yields $\mathbf{D}_i = (m-1)\left(\mathbf{H}_i - \frac{1}{m}\right)$. Similarly, we can prove that the cross-leverage scores are equal to

$$\mathbf{H}_{ij} = \frac{1}{m} + \frac{1}{m-1}\mathbf{X}_{k_{i*}}\mathbf{\Sigma^{-1}}\mathbf{X}_{k_{j*}}. \tag{10}$$

To prove the relationship between $\mathbf{H}_{ij}$ and $\mathbf{D}_{ij}$ we observe that

$$\begin{aligned}
\mathbf{D}(\mathbf{X}_{k_{i*}}, \mathbf{X}_{k_{j*}}) &= (\mathbf{X}_{k_{i*}} - \mathbf{X}_{k_{j*}}^\top)\mathbf{\Sigma}^{-1}(\mathbf{X}_{k_{i*}} - \mathbf{X}_{k_{j*}}) \\
&= \mathbf{D}(\mathbf{X}_{k_{i*}}, 0) + \mathbf{D}(\mathbf{X}_{k_{j*}}, 0) - 2\mathbf{X}_{k_{i*}}\mathbf{\Sigma}^{-1}\mathbf{X}_{k_{j*}} \\
&= (m-1)(\mathbf{H}_i - \frac{1}{m}) + (m-1)(\mathbf{H}_j - \frac{1}{m}) - 2(m-1)(\mathbf{H}_{ij} - \frac{1}{m}) \\
&= (m-1)(\mathbf{H}_i + \mathbf{H}_j - 2\mathbf{H}_{ij}).
\end{aligned}$$

Taking $\mathbf{X}_{k_{i*}} = \mathbf{X}_{k_{j*}}$ we get $\mathbf{D}(\mathbf{X}_{k_{i*}}, \mathbf{X}_{k_{j*}}) = 0$. Thus, we have shown that Mahalanobis distance between two vectors can be computed using the corresponding vector's leverage scores.

One of the key computational bottlenecks of the Mahalanobis distance is the computation of the inverse of the SNP covariance matrix $\mathbf{G}$, as required in Equation 3. In real datasets, with the improvements in genotyping and sequencing technologies, the number of SNPs can be in the millions, thereby making $\mathbf{G}$ impossible to invert. Here, we propose to approximate the Mahalanobis distance by computing leverage and cross-leverage scores in a fast and efficient way. As we have shown in Equations 10 and 7, following up on previous work [20], the Mahalanobis distance can be written in terms of leverage scores. Advances in the theoretical computer science community have resulted in faster algorithms to compute leverage and cross-leverage scores. Hence, we can compute approximation to these scores using random sampling algorithms that come with provable theoretical guarantees [6]. For our purposes, we work with simulated data as described above for 1,000 individuals and 500,000 SNPs, which could be processed efficiently in commodity hardware.

Fast computation of standard errors

The standard error of the estimates in order to calculate the p-values associated with each marker to compute the significance of its association with the trait. The standard error for each marker i in ridge regression is given by

$$SE(\hat{\beta}_i^{ridge}) = \frac{\sigma}{\nu}\left\|(\mathbf{X}\mathbf{X}^\top + \lambda\mathbf{I}_m)^{-1}\mathbf{X}_{*i}\right\|_2. \tag{11}$$

Recall that $\mathbf{X}_{*i}$ is the i-th column of $\mathbf{X}$ and ν is known as the residual degrees of freedom. We set ν as shown in previous work [11] to the following,

$$\nu = m - c\mathbf{X}(\mathbf{X}^\top\mathbf{X} + \lambda\mathbf{I})^{-1}\mathbf{X}^\top, \tag{12}$$

for a small constant $c > 0$.

For biobank-scale data-sets requiring terabytes of memory, computing the standard error can be a challenge. However, we can use random projection based sketching matrices to approximate standard errors for each marker by reducing the dimensionality of the genotype matrix $\mathbf{X}$ using a sketching matrix $\mathbf{S} \in \mathbb{R}^{n \times r}$ to form a sketch $\mathbf{XS}$. We can rewrite the standard error in Equation 11 as follows:

$$\widetilde{\widehat{SE}}(\hat{\beta}_i) = \sigma^2 \| \left(\mathbf{XSS}^\top \mathbf{X}^\top + \lambda \mathbf{I}_m\right)^{-1} \mathbf{X}^{(i)} \|_2^2. \tag{13}$$

The sketched matrix $\mathbf{XS}$ has the same rank but much fewer columns than $\mathbf{X}$, satisfying $r \ll n$. Sketching, in general, is used to speed up many numerical linear algebra operations [5, 7, 9]. The sketching dimension r directly affects the accuracy of the approximate standard errors. Some prior knowledge of the design matrix $\mathbf{X}$ helps determine the target rank r in order to get satisfactory error guarantees. The sketching matrix $\mathbf{S}$ can be chosen simply to be i.i.d normal random variables with mean equal to zero and variance equal to $\frac{1}{r}$. There exist other ways to construct $\mathbf{S}$ [1, 4, 7, 8].

Supplementary Results

Simulated Data

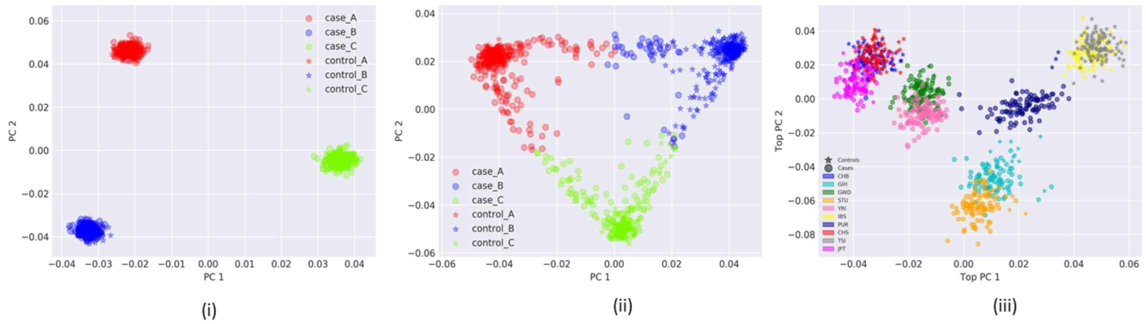

Supplementary Figure 1: Projection of the samples from three populations simulated from (i) BN (ii) PSD ($\alpha = \{0.1, 0.1, 0.1\}$) and (iii) TGP model on the top two principal components.

The BN model simulates scenarios with unrelated isolated populations (Figure 1 (i)) and serves as our base case for arbitrarily structured population with no admixture. Projecting the samples on the top two PCs clearly shows three isolated clusters with no connections between them. This is the ideal case where the populations are not mixing due to environmental factors acting as barriers of gene flow between them. GWAS has shown to be robust in these settings [19]; however, cryptic relatedness within cluster remains a p issue [2]. We compared these methods on all 100 scenarios with the p-value threshold set to 10^{-5}. We used GCTA tools [22] to simulate binary traits with 20% of the individuals as cases and enforcing 100 of the SNPs to be causal with heritability set to 0.5.

The Armitage trend χ^2 test with no population structure correction renders many of the SNPs in the simulation study as true associations, resulting in more spurious associations, clearly highlighting the need for population structure correction. PCA or LMM based approaches on

the other hand return roughly the expected number of spurious associations as also shown in prior work [17]. CluStrat results in an increase over standard approaches in detecting causal variants. The Armitage trend χ^2 test has the maximum number of causal associations, but also captures the most spurious associations. CluStrat outperforms all other standard methods for population stratification correction in this scenario while still detecting no spurious associations (Supplementary Figures 2, 3, and 4). This shows that in the ideal case of population structure correction, CluStrat can identify more causal SNPs due to the structure-informed clustering.

The PSD model emulates real world datasets more closely than the BN model. It allows for admixed individuals and gradients across the populations. It is sampled from the Dirichlet distribution, parameterized by a concentration parameter $\alpha \in \mathbb{R}^d$. We set d to three, which denotes the number of ancestral populations for all the simulations that we conducted. The TGP model closely approximates real-world allele frequency distribution. CluStrat was able to identify more causal SNPs in these more nuanced settings due to leveraging the structure-informed clustering.

The UKBB model generates synthetic data from two ancestral backgrounds, Irish and British, using a "mosaic-chromosome" scheme modified from [12, 13]. The general concept is to take a small set of individuals that are genetically distinct and generate artificial individuals by sampling their genomes. We began by selecting all individuals with British and Irish ancestries from the UK Biobank data after performing quality control and pruning, thus resulting in a dataset of 435,655 individuals and 265,642 SNPs. Unlike the other simulation models that use Fst measures to create distinct populations, the UKBB model filters the samples based on their ancestries inferred from SNP data (using the top two PCs) to ensure that the two groups were genetically distinct. We selected 100 samples from that subset of individuals to treat as the founders or ancestors to generate the artificial individuals from. We divided the genome into consecutive segments of 2,000 variants and generated *unrelated* individuals by selecting each segment from one of the 100 ancestors chosen at random and simulated *related* individuals by selecting the segments from a smaller number of ancestors according to the degree of relatedness. This process is done for both the Irish and British populations. Finally, we used GCTA tools [22] to simulate quantitative and binary traits for our simulated individuals.

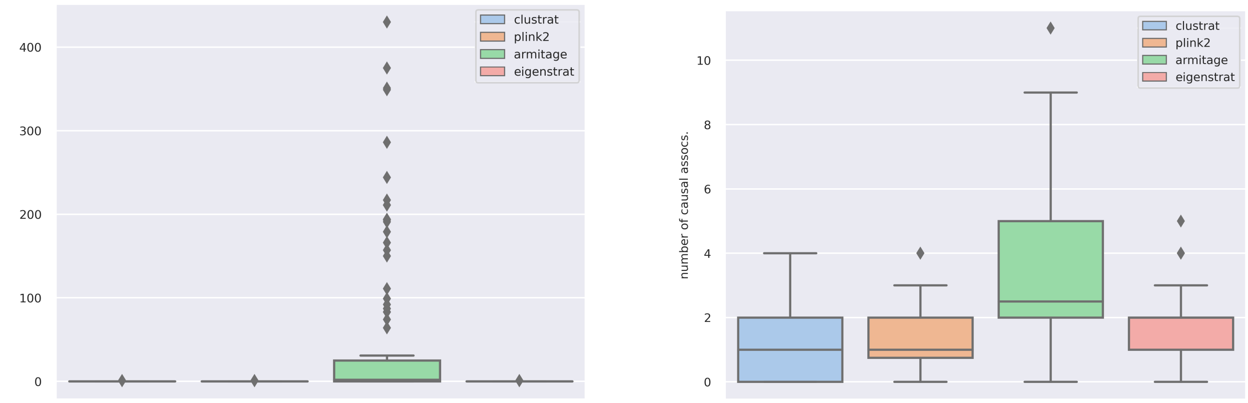

(a) Spurious associations (b) Causal associations

Supplementary Figure 2: Box plots for spurious and causal associations on the BN model using the Armitage trend χ^2 statistic, PLINK2, Eigenstrat, and CluStrat.

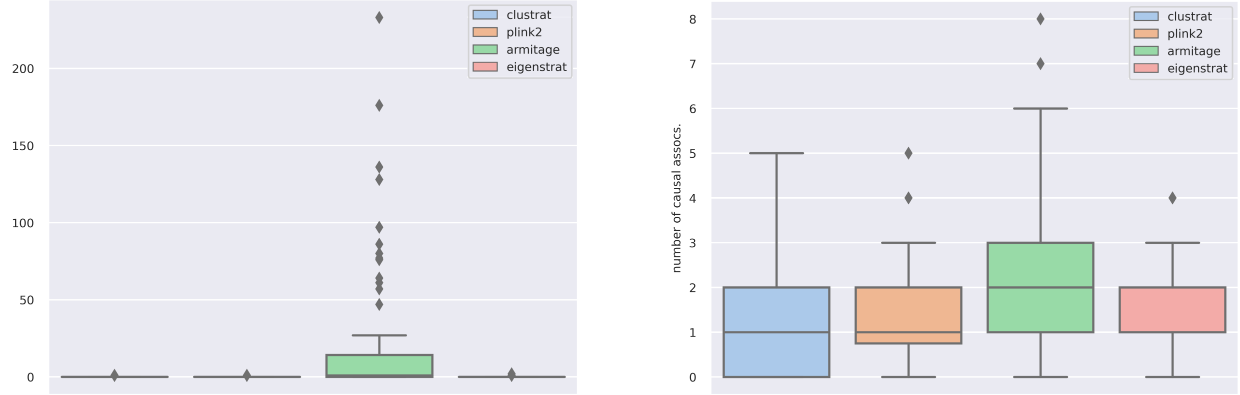

(a) Spurious associations (b) Causal associations

Supplementary Figure 3: Box plots for spurious and causal associations on the PSD model using the Armitage trend χ^2 statistic, PLINK2, Eigenstrat, and CluStrat.

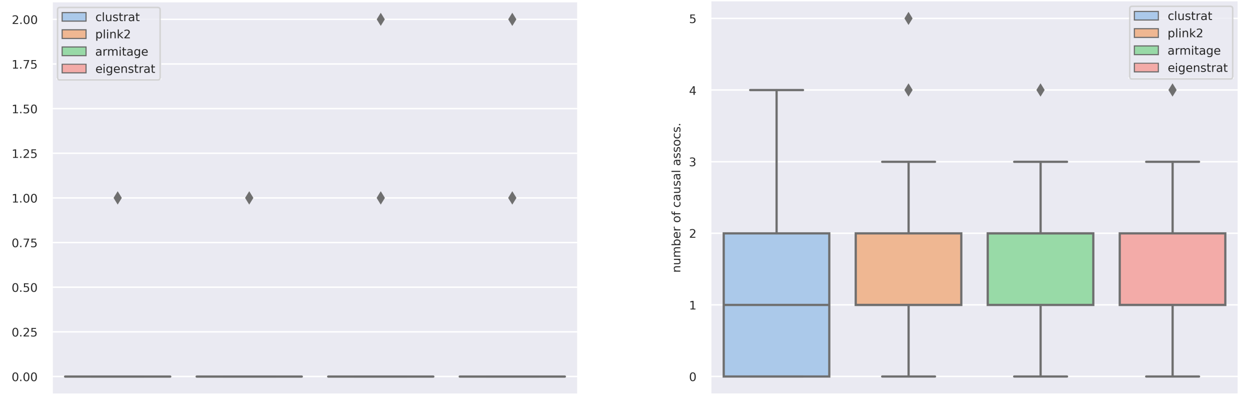

(a) Spurious associations (b) Causal associations

Supplementary Figure 4: Box plots for spurious and causal associations on the TGP model using the Armitage trend χ^2 statistic, PLINK2, Eigenstrat, and CluStrat.

Comparing distance metrics

CluStrat with the Euclidean distance metric based GRM also uses structural information as part of the relationships between the individuals within and between population groups. The GRM with the Euclidean distance metrix is straightforward to compute: $\mathbf{D} = \mathbf{X}^\top\mathbf{X}$, where $\mathbf{X} \in \mathbb{R}^{n\times m}$ is the genotype matrix (n markers and m samples). We show that although Euclidean distances between individuals are straightforward to compute, they fail to distinguish fine-grained relationships between individuals in the same cluster due to cryptic relatedness. This is highlighted by performing AHC using Ward's linkage method, which minimizes the increase in the sum of squares between two cluster centroids, in order to determine when to merge two clusters (Supplementary Figure 6). Using the Mahalanobis distance based GRM is instead of Euclidean distance in AHC on the PSD model with 1,000 individuals and 10,000 SNPs across three admixed arbitrarily structured ethnic groups, reveals four broad clusters with various fine-grained subclusters. The Mahalanobis distance helps recover cryptic relatedness and substructure within a

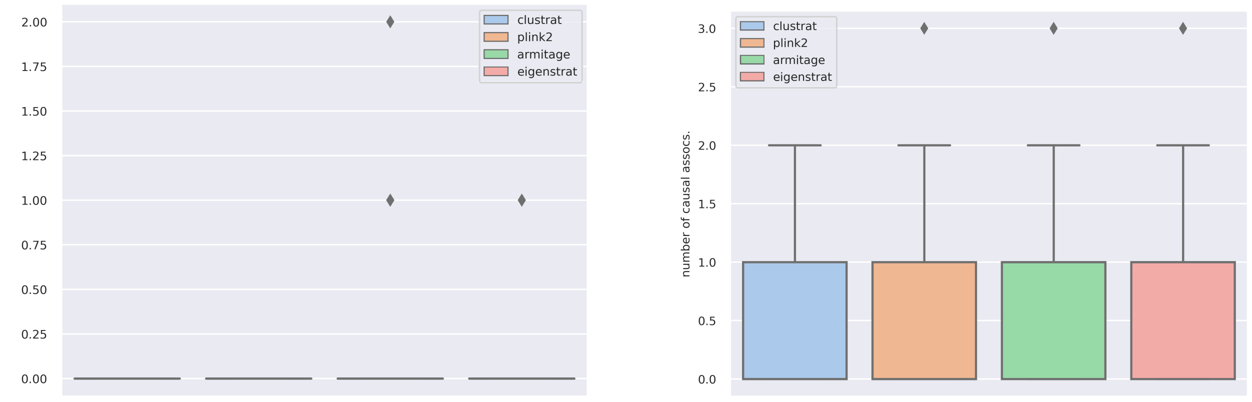

(a) Spurious associations (b) Causal associations

Supplementary Figure 5: Box plots for spurious and causal associations on the UKBB model using the Armitage trend χ^2 statistic, PLINK2, Eigenstrat, and CluStrat.

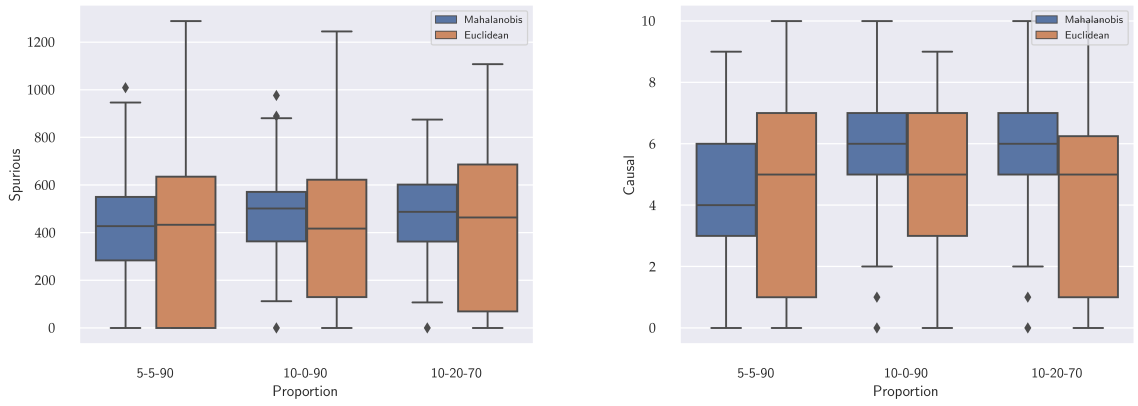

(a) Spurious associations (b) Causal associations

Supplementary Figure 6: Box plots for spurious and causal associations obtained by running AHC with Mahalanobis and Euclidean distances on the PSD model ($\alpha = \{0.1, 0.1, 0.1\}$). We observe similar performance for both distance metrics in terms of identifying true causal variants. However, the Mahalanobis distance discovers far less spurious associations than the Euclidean distance.

population. Due to admixture in the PSD model, the dendrogram finds three broad clusters. It subsequently finds different subclusters at different depths on the horizontal axis, therefore identifying the interaction between individuals inside a cluster. This is a significant advantage of using the Mahalanobis distance instead of the Euclidean distance, as the latter only reveals three broad clusters with indistinguishable interactions within each cluster (Figure 6). We note that as we increase the scale of admixed genotype data with more complex structure, the Mahalanobis distance is better suited as it is known to project correlated high dimensional data to an uncorrelated lower dimensional space where it recovers the hidden Euclidean distances [14].

Real Data

Height GWAS

We used 18,698 highly-related individuals in the UK Biobank cohort (first degree or higher according to the kinship coefficient) with 44,818 SNPs, related to the largest effect sizes in relation to height, from summary statistics data generated by [21]. CluStrat was able to correct for the previously uncorrected population structure when compared with Eigenstrat or GEMMA with a negative slope of 0.079 and a negligible Spearman $r = 0.032$. We note here that although CluStrat without any multiple hypothesis correction does a good job in correcting for population structure (Supplementary Figure 7), the slope is negative when compared to other methods as well as no correction. But, with Bonferroni correction of the p-values obtained by CluStrat we achieve better performance of correction as well as invert the direction of the slope to match other methods (Figure 2). Thus, correcting for multiple hypotheses in CluStrat is essential to control for false positives and it produces better result in correcting for underlying cryptic relatedness confounding of height GWAS in UKBB.

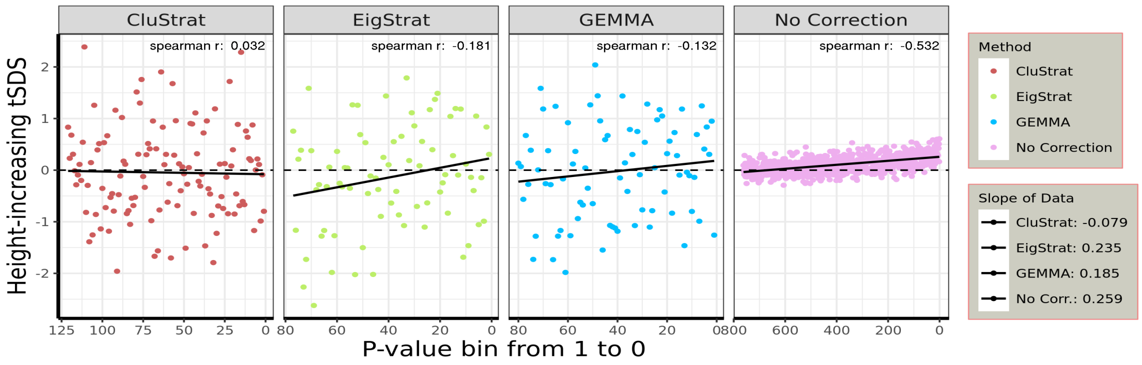

Supplementary Figure 7: tSDS for height-increasing alleles in the UK Biobank subset using CluStrat, the PCA-based Eigenstrat method, and the LMM-based GEMMA method. SNPs are ordered by p-value (in bins of 50 in the 'No correction' scenario). The dashed line indicates null-expectation and the black line is the linear regression fit.

SCZ data

SCZ data was available from the Wellcome Trust Case Control Consortium (WTCCC2) study containing 5,893 individuals (5,416 SCZ controls and 477 cases) with 18,683 markers. We first retained high quality imputed SNPs with INFO > 0.3. We performed quality control (QC) with PLINK [3] with removing missing samples and variants with 5% missing values, minor allele frequency (MAF) < 0.05, Hardy-Weinberg Equilibrium (HWE) $< 10^{-6}$ and removeing samples with more than 3 standard deviations in heterozygosity rates and closely related individuals $pi - hat > 0.125$ and pruning for LD between variants with $r^2 = 0.2$ and ended up with 18,683 markers.

AMI data

We applied CluStrat on AMI data from the UK Biobank with 23,142 individuals (11,610 controls and 11,532 cases) and 784,256 imputed genotypes. We first retained high quality SNPs with INFO

> 0.3. We performed quality control (QC) with PLINK [3] with removing missing samples and variants with 5% missing values, minor allele frequency (MAF) < 0.05, Hardy-Weinberg Equilibrium (HWE) $< 10^{-6}$ and removeing samples with more than 3 standard deviations in heterozygosity rates and closely related individuals $pi - hat > 0.125$ and pruning for LD between variants with $r^2 = 0.9$ and ended up with 208,337 markers.

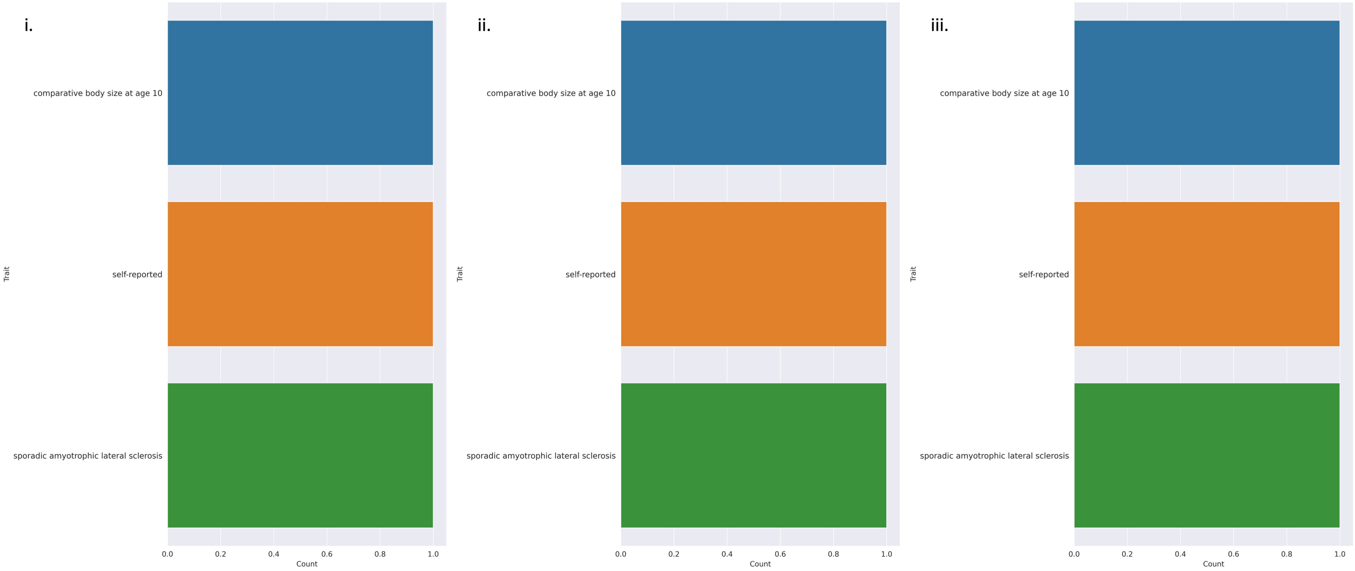

Supplementary Figure 8: Mapped GWAS catalog traits for Schizophrenia dataset pruned at varying levels: 0.2 (i), 0.9 (ii), no pruning (iii).

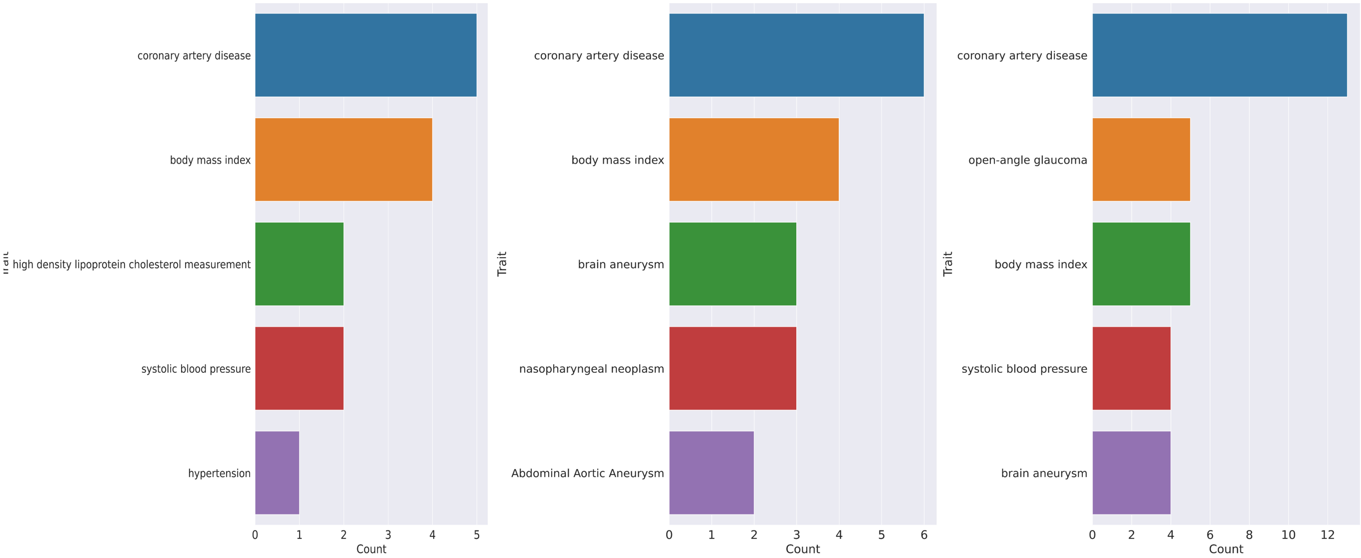

Supplementary Figure 9: Top 5 GWAS catalog traits with the most matching variants for Acute Myocardial Infarction dataset pruned at varying levels: 0.2 (i), 0.9 (ii), no pruning (iii).

References

[1] Dimitris Achlioptas. Database-friendly random projections: Johnson-lindenstrauss with binary coins. *Journal of computer and System Sciences*, 66(4):671–687, 2003.

[2] Jeremy J Berg, Arbel Harpak, Nasa Sinnott-Armstrong, Anja Moltke Joergensen, Hakhamanesh Mostafavi, Yair Field, Evan August Boyle, Xinjun Zhang, Fernando Racimo, Jonathan K Pritchard, et al. Reduced signal for polygenic adaptation of height in uk biobank. *eLife*, 8:e39725, 2019.

[3] Christopher C. Chang, Carson C. Chow, Laurent CAM Tellier, Shashaank Vattikuti, Shaun M. Purcell, and James J. Lee. Second-generation plink: rising to the challenge of larger and richer datasets. *GigaScience*, 4(1):7, 2015.

[4] Moses Charikar, Kevin Chen, and Martin Farach-Colton. Finding frequent items in data streams. In *International Colloquium on Automata, Languages, and Programming*, pages 693–703. Springer, 2002.

[5] Kenneth L Clarkson and David P Woodruff. Low-rank approximation and regression in input sparsity time. *Journal of the ACM (JACM)*, 63(6):54, 2017.

[6] Petros Drineas, Malik Magdon-Ismail, Michael W Mahoney, and David P Woodruff. Fast approximation of matrix coherence and statistical leverage. *Journal of Machine Learning Research*, 13(Dec):3475–3506, 2012.

[7] Petros Drineas, Michael W Mahoney, and Shan Muthukrishnan. Sampling algorithms for l 2 regression and applications. In *Proceedings of the seventeenth annual ACM-SIAM symposium on Discrete algorithm*, pages 1127–1136. Society for Industrial and Applied Mathematics, 2006.

[8] Petros Drineas, Michael W Mahoney, and Shan Muthukrishnan. Relative-error cur matrix decompositions. *SIAM Journal on Matrix Analysis and Applications*, 30(2):844–881, 2008.

[9] Petros Drineas, Michael W Mahoney, Shan Muthukrishnan, and Tamás Sarlós. Faster least squares approximation. *Numerische mathematik*, 117(2):219–249, 2011.

[10] Wei Hao, Minsun Song, and John D Storey. Probabilistic models of genetic variation in structured populations applied to global human studies. *Bioinformatics*, 32(5):713–721, 2015.

[11] Trevor J Hastie. Generalized additive models. In *Statistical models in S*, pages 249–307. Routledge, 2017.

[12] L Jiang, Z Zheng, and H Fang. A generalized linear mixed model association tool for biobank-scale data. *Nature Genetics*, 2021.

[13] Po-Ru Loh. Efficient bayesian mixed-model analysis increases association power in large cohorts. *Nature Publishing Group*, 47, 2015.

[14] Prasanta Chandra Mahalanobis. On the generalized distance in statistics. In *Proceedings of the National Institute of Science of India*. National Institute of Science of India, 1936.

[15] Boby Mathew, Jens Léon, and Mikko J Sillanpää. A novel linkage-disequilibrium corrected genomic relationship matrix for snp-heritability estimation and genomic prediction. *Heredity*, 120(4):356, 2018.

[16] Ann FS Mitchell and Wojtek J Krzanowski. The mahalanobis distance and elliptic distributions. *Biometrika*, 72(2):464–467, 1985.

[17] Alkes L Price, Nick J Patterson, Robert M Plenge, Michael E Weinblatt, Nancy A Shadick, and David Reich. Principal components analysis corrects for stratification in genome-wide association studies. *Nature genetics*, 38(8):904, 2006.

[18] Minsun Song, Wei Hao, and John D Storey. Testing for genetic associations in arbitrarily structured populations. *Nature genetics*, 47(5):550, 2015.

[19] Peter M. Visscher, Naomi R. Wray, Qian Zhang, Pamela Sklar, Mark I. McCarthy, Matthew A. Brown, and Jian Yang. 10 years of gwas discovery: Biology, function, and translation. *The American Journal of Human Genetics*, 101(1):5 – 22, 2017.

[20] Irving B Weiner. *Handbook of psychology, history of psychology*, volume 1. John Wiley & Sons, 2003.

[21] Andrew R Wood, Tonu Esko, Jian Yang, Sailaja Vedantam, Tune H Pers, Stefan Gustafsson, Audrey Y Chu, Karol Estrada, Zoltán Kutalik, Najaf Amin, et al. Defining the role of common variation in the genomic and biological architecture of adult human height. *Nature genetics*, 46(11):1173–1186, 2014.

[22] Jian Yang. Gcta: a tool for genome-wide complex trait analysis. *American journal of human genetics*, 88:76–82, January 2011.
